# Clinical characteristics and outcomes of neonates with polymicrobial ventilator-associated pneumonia in the intensive care unit

**DOI:** 10.1186/s12879-021-06673-9

**Published:** 2021-09-17

**Authors:** Hsiao-Chin Wang, Ming-Horng Tsai, Shih-Ming Chu, Chen-Chu Liao, Mei-Yin Lai, Hsuan-Rong Huang, Ming-Chou Chiang, Ren-Huei Fu, Jen-Fu Hsu

**Affiliations:** 1 Division of Pediatric Neonatology, Department of Pediatrics, Chang Gung Memorial Hospital, Taoyuan, Taiwan; 2 Division of Neonatology and Pediatric Hematology/Oncology, Department of Pediatrics, Chang Gung Memorial Hospital, Yunlin, Taiwan; 3 College of Medicine, Chang Gung University, Taoyuan, Taiwan; 4 Division of Pediatric Pulmonology, Department of Pediatrics, Chang Gung Memorial Hospital, Taoyuan, Taiwan; 5 Division of Neonatology, Department of Pediatrics, Chang Gung Memorial Hospital, No. 5, Fu-Shin Rd., Kwei Shan, Taoyuan County, 333, Linkou, Taiwan, ROC

**Keywords:** Ventilator-associated pneumonia, Respiratory failure, Neonates, Multidrug resistant pathogens, Broad-spectrum antibiotics

## Abstract

**Background:**

Ventilator associated pneumonia (VAP) caused by more than one microorganisms is not uncommon and may be potentially challenging, but the relevant data is scarce in ventilated neonates. We aimed to investigate the clinical characteristics and outcomes of polymicrobial VAP in the neonatal intensive care unit (NICU).

**Methods:**

All neonates with definite diagnosis of VAP from a tertiary level neonatal intensive care unit (NICU) in Taiwan between October 2017 and September 2020 were prospectively observed and enrolled for analyses. All clinical features, therapeutic interventions and outcomes were compared between the polymicrobial VAP and monomicrobial VAP episodes. Multivariate regression analyses were used to find the independent risk factors for treatment failure.

**Results:**

Among 236 episodes of neonatal VAP, 60 (25.4%) were caused by more than one microorganisms. Polymicrobial VAP episodes were more likely to be associated with multidrug-resistant pathogens (53.3% versus 34.7%, P = 0.014), more often occurred in later days of life and in neonates with prolonged intubation and underlying bronchopulmonary dysplasia. Otherwise most clinical characteristics of polymicrobial VAP were similar to those of monomicrobial VAP. The therapeutic responses and treatment outcomes were also comparable between these two groups, although modification of therapeutic antibiotics were significantly more common in polymicrobial VAP episodes than monomicrobial VAP episodes (63.3% versus 46.2%; P < 0.001). None of any specific pathogens was significantly associated with worse outcomes. Instead, it is the severity of illness, including presence of concurrent bacteremia, septic shock, and requirement of high-frequency oscillatory ventilator and underlying neurological sequelae that are independently associated with treatment failure.

**Conclusions:**

Polymicrobial VAP accounted for 25.4% of all neonatal VAP in the NICU, and frequently occurred in neonates with prolonged intubation and underlying bronchopulmonary dysplasia. In our cohort, most clinical features, therapeutic responses and final outcomes of neonates with monomicrobial and polymicrobial VAP did not differ significantly.

**Supplementary Information:**

The online version contains supplementary material available at 10.1186/s12879-021-06673-9.

## Introduction

Ventilator-associated pneumonia (VAP) accounts for one-fifth to one-fourth of healthcare associated infections (HAIs) in the neonatal intensive care unit (NICU), and is very difficult to have the confirmed diagnosis [1, 2]. VAP is confirmed in approximately 15–20% of critically ill/premature neonates who have intubation for more than 48 h [1–4], and the reported mortality rate was around 9.3–16.4% in recent studies [5–7]. Although the causative pathogens are associated with the hospital course and duration of mechanical ventilation, gram-negative bacilli account for the majority of VAP in the NICU [6–9]. VAP is associated with significant morbidity due to prolonged requirement of ventilation and an increased risk of subsequent another episode of HAI [8, 9]. In addition, increased hospital costs and resource use are also noted in neonates with VAP [9–11].

Previous studies in both adult and pediatric ICUs found 25–40% of all VAP episodes are caused by more than one microorganism [9, 12–14]. Polymicrobial VAP is supposed to cause therapeutic challenge, especially when neonates have previous broad-spectrum antibiotic exposure or endotracheal colonization of multidrug-resistant (MDR) pathogens [15, 16]. Currently no relevant studies have been conducted to investigate the demographics and clinical outcomes of neonates developing polymicrobial lung infection. In addition, previous studies are limited by retrospective design and sometimes uncertain cases of colonization or true infection [1, 2, 9]. Therefore, to ascertain the epidemiologic aspects and clinical features of polymicrobial VAP in neonates, we conducted a prospective study with consecutive neonates receiving intubation for ≥ 48 h, in whom VAP was confirmed by strict diagnostic criteria of the Centers for Disease Control and Prevention (CDC) [1, 17].

## Methods

### Patients, study design and setting

Between October 2017 and September 2020, all neonates with mechanical intubation for more than 48 h in the NICUs of Chang Gung Memorial Hospital (CGMH) were prospectively observed and followed if they had clinical symptoms and signs of VAP. The NICUs of CGMH contain a total of three units. The total capacity is 49 beds equipped with ventilator and 58 beds of special care nurseries. This study was supported by the CGMH research foundation to investigate the clinical application and diagnostic accuracy of nonbronchoscopic bronchoalveolar lavage (NBBAL) in neonatal VAP. The standard endotracheal aspiration for culture of the aspirate samples was performed in all neonates with clinically suspected VAP. However, all these patients were randomized and only some of them received NBBAL examination [18]. We used the NBBAL procedure because it is applicable in extremely preterm neonates and has been proven safe and high diagnostic accuracy [18, 19]. This study was approved by the Institutional Review Board of CGMH, and written informed consent was obtained from parents of the neonates prior to be enrolled in this study.

### Definition

The updated diagnostic criteria of the CDC for neonatal VAP (Additional file 1: Table S1) was applied in this study [1, 17]. All neonates with clinically suspected VAP who fulfilled the clinical, radiological, and laboratory, and microbiological criteria of VAP were enrolled and prospectively observed. Neonates who did not received NBBAL and only sputum cultures were available were enrolled because the CDC diagnostic criteria permit the diagnosis of “Clinically defined pneumonia” which is based only on clinical features and radiological images without documentation of any isolated pathogen [1, 19]. Polymicrobial VAP was defined as more than one pathogenic microorganism identified from a single set of sputum culture. Only the first episode of VAP in each patient was considered in the analysis.

We defined the use of ampicillin/sulbactam or oxacillin plus gentamicin or ceftriaxone as first-line antibiotics [20]. Resistance to first-line antibiotics was considered when the causative microorganism was resistant or one of the isolated bacterial strains in polymicrobal VAP cases was resistant to one of these antibiotics. Antibiotic susceptibility patterns were determined according to methods recommended by the National Committee for Clinical Laboratory Standards Institute (CLSI) for the disk diffusion method and categorical assignment was performed using CLSI breakpoints [21]. Multidrug-resistant (MDR) pathogens were defined as the bacterial strain is resistant to at least one agent in three or more of the following antimicrobial categories: carbapenems (imipenem and meropenem); penicillins (piperacillin, ticarcillin and piperacillin/tazobactam); broad-spectrum cephalosporins (ceftazidime and cefepime); monobactams (aztreonam); aminoglycosides; and fluorquinolones [22, 23].

All comorbidities of prematurity, including pulmonary hypertension, respiratory distress syndrome, neurological sequelae, bronchopulmonary dysplasia (BPD), necrotizing enterocolitis, short bowel syndrome and periventricular leukomalacia were based on the latest updated diagnostic criteria in the standard textbook of neonatology [24]. Onset of VAP was defined when the chest X-ray imaging was performed and endotracheal aspirates and/or NBBAL was performed. Inappropriate empirical antibiotics were considered when one of the bacteria strains was resistant to the initial empirical treatment [25].

### Data collection

Patient demographics, clinical parameters at VAP onset, therapeutic interventions, responses to treatment and outcomes were prospectively collected for all neonates with clinically diagnosed VAP. The empiric antibiotics depended on the decision of attending physician, but therapeutic antibiotics usually will be modified according to the culture results. Severity of illness was evaluated at the onset of each VAP episode using the neonatal therapeutic intervention scoring system (NTISS) [26]. All patients were followed until discharge or death. The case was excluded if the patient was transferred to other hospital and the final outcome was unknown. The administration of any antibiotics for at least three days during the one month preceding the VAP event was defined as antibiotic exposure and recorded.

### Statistical analysis

Parametric variables are expressed as mean (standard deviation, SD) and continuous variables with non-parametric distributions are expressed as median (interquartile range, IQR). Comparisons between continuous variables of two subgroups were analyzed using paired Student’s t-test and the paired Wilcoxon rank sum tests. Categorical variables were compared with Chi-square tests or Fisher’s exact tests. All *p*-values were two tailed, and *p*-values < 0.05 were considered to be statistically significant. All statistical analysis was performed using SPSS (version 21.0; IBM, Armonk, NY).

We considered all potential microorganisms isolated from the culture of NBBAL fluid regardless of the number of CFU per milliliter as the causative pathogens of VAP. Subgroup analyses were performed between monomicrobial VAP episode and polymicrobial VAP episode. The primary outcomes were VAP attributable mortality and final in-hospital mortality. Because we aimed to investigate the impacts of therapeutic antibiotics on the outcomes, the secondary outcome was treatment failure of the VAP episodes. Treatment failure of VAP included neonates who died directly due to VAP, those required therapeutic antibiotics for more than two weeks, progression to bacteremia, and clinical deterioration occurred even after 7-days effective antibiotic treatment. Therefore, risk factors of treatment failure in neonatal VAP were identified using univariate and multivariate logistic regression analyses. All variables with P values < 0.1 were enrolled into the multivariate logistic regression model.

## Results

### Epidemiology of VAP and the microorganisms

During the study period, 236 (28.0%) of 844 critically ill or preterm neonates who had intubation for more than 48 h were found to have experienced at least one VAP episode during hospitalization. The median (interquartile range, IQR) gestational age (GA) and birth body weight (BBW) of this cohort were 26.0 (25.0–28.0) weeks and 871.0 (720.0–1080.0) g, respectively. Significantly more male patients than female patients were noted (a male/female ratio of 1.51:1) in this cohort. These patients had a total of 27,284 neonates-ventilator days, and the incidence rate of VAP was 8.7 episodes/per 1000 neonates-ventilator days. All these VAP cases were prospectively followed until discharge or death. The median (IQR) time of VAP onset was 30.0 (19.0–48.5) days of life.

Among the 236 patients for whom the definite diagnosis of VAP had been confirmed, 176 (74.6%) had monomicrobial infections and 60 (25.4%) had at least two bacteria isolated from pulmonary specimens. In polymicrobial VAP, 54 (90.0%) had two bacteria present and 6 (10.0%) had three bacteria present. A total of 302 bacteria were grown in cultures at significant concentrations for the 236 episodes of VAP. Gram-negative bacilli and gram-positive cocci accounted for 215 (71.2%) and 87 (28.8%) of isolated bacteria, respectively. The most common pathogenic microorganism spectra in this series were *Staphylococcus aureus* (81 [26.8%]), followed by *K. pneumonia* (42 [13.9%]), *Pseudomonas aeruginosa* (33 [10.9%]), *Acinetobacter baumannii* (32 [10.6%]), *E coli* (24 [7.9%]) and *Serratia marcescens* (21 [7.0%]). 93 (39.4%) of all neonatal VAP episodes were caused by MDR pathogens, and significantly more polymicrobial VAP episodes were caused by MDR pathogens (53.3% vs. 34.7%, P = 0.014) (Table 1). All fungal strains identified in this cohort were interpreted as colonization and were not treated with antifungal agents.

**Table 1:** Pathogens distribution of neonatal ventilator-associated pneumonia (VAP) in the NICU of CGMH, October 2017 to September 2020

	Polymicrobial VAP (total n = 60 episodes)	Monomicrobial VAP (total n = 176 episodes)	MDR pathogens (total n = 98 bacteria)
Gram-positive cocci	41 (32.5)	44 (25.0)	35 (35.7)
* Methicillin resistant Staphylococcus aureus*	20 (15.9)	15 (8.5)	35 (35.7)
* Methicillin sensitive Staphylococcus aureus*	18 (14.3)	28 (15.9)	0 (0)
Enterococcus spp.	1 (0.8)	1 (0.6)	0 (0)
* Group B Streptococcus*	2 (1.6)	0 (0)	0 (0)
Gram-negative bacilli	85 (67.5)	122 (69.3)	59 (60.2)
* Klebsiella pneumonia*	21 (16.7)	21 (11.9)	13 (13.3)
* Pseudomonas aeruginosa*	11 (8.7)	21 (11.9)	6 (6.1)
* Acinetobacter baumannii*	15 (11.9)	17 (9.7)	4 (4.1)
*Escherichia coli*	9 (10.9)	15 (8.5)	11 (11.2)
* Serratia marcescens*	7 (7.1)	14 (8.0)	0 (0)
* Enterobacter spp.*	2 (1.6)	19 (10.8)	2 (2.0)
* Stenotrophomonas maltophilia*	12 (9.5)	6 (3.4)	18 (18.4)
* Klebsiella aerogenes*	5 (4.0)	6 (3.4)	3 (3.1)
* Klebsiella oxytoca*	3 (2.4)	3 (1.7)	2 (2.0)
Others^a^	0 (0)	10 (5.7)	4 (4.1)

*MDR pathogens* multidrug-resistant pathogens

^a^Including *Burkholderia cepacia* (3), *Corynebacterium striatum* (2), *Morganella species* (1), *Citrobacter koseri* (2), *Moraxella catarrhalis* (1) and *Hemophilus influenzae* (1)

### Comparisons between monomicrobial VAP and polymicrobial VAP

The patients’ demographics, underlying chronic comorbidities and clinical features of all VAP episodes are presented in Table 2. Most VAP episodes (197/236, 83.5%) occurred in neonates with underlying chronic comorbidities, and 105 (44.5%) occurred in neonates with multiple chronic comorbidities. The polymicrobial VAP episodes did not significantly differ from monomicrobial VAP episodes in terms of most patient demographics, clinical features, and severity of illness (by the NTISS score at onset of VAP) (Table 2). However, polymicrobial VAP episodes were more likely to occur in later days of life (median 39.5 versus 26.5 days old, P < 0.001) and in neonates with prolonged intubation and underlying bronchopulmonary dysplasia (86.7% vs. 63.1%, P = 0.001) when compared with monomicrobial VAP episodes. Previous antibiotic exposure (within one month before VAP) and previous bacteremia were also significantly more common in neonates with polymicrobial VAP. Furthermore, 37.7% (n = 89) of all VAP episodes occurred in neonates who were on antibiotic treatment for previous episode of nosocomial infections, especially more common in polymicrobial VAP episodes.

**Table 2:** Patient demographics, characteristics, and clinical presentation of monomicrobial ventilator-associated pneumonia (VAP) versus polymicrobial VAP in CGMH, October 2017-March 2020

Characteristics	All VAP episodes (total n = 236)	Monomicrobial VAP episodes (total n = 176)	Polymicrobial VAP episodes (total n = 60)	P values
Cases demographics				
Gestational age (weeks), median (IQR)	26.0 (25.0–28.0)	26.0 (25.0–28.8)	26.0 (25.0–28.0)	0.984
Birth weight (g), median (IQR)	871.0 (720.0–1080.0)	870.5 (720.0–1090.0)	876.0 (730–1097.0)	0.856
Gender (male/female), n (%)	142 (60.2)/94 (38.8)	105 (59.7)/71 (40.3)	37 (61.7)/23 (38.3)	0.879
5 min Apgar score ≤ 7, n (%)	78 (33.1)	55 (31.3)	23 (38.3)	0.719
Inborn/outborn, n (%)	208 (88.1)/28 (11.9)	154 (87.5)/22 (12.5)	54 (90.0)/6 (10.0)	0.817
Birth by NSD/Cesarean section, n (%)	93 (39.4)/143 (60.6)	65 (36.9)/111 (63.1)	28 (46.7)/32 (53.3)	0.221
Respiratory distress syndrome (≥ Gr II), n (%)	165 (69.9)	122 (69.3)	43 (71.7)	0.752
Intraventricular hemorrhage (≥ Stage III), n (%)	19 (8.1)	14 (8.0)	5 (8.3)	0.800
Underlying chronic comorbidities, n (%)				
Neurological sequelae	69 (29.4)	56 (31.8)	13 (21.7)	0.142
Bronchopulmonary dysplasia	163 (69.1)	111 (63.1)	52 (86.7)	0.001
Cardiovascular diseases	35 (14.8)	27 (15.3)	8 (13.3)	0.835
Gastrointestinal sequelae	67 (28.4)	48 (27.3)	19 (31.7)	0.512
Renal disorders	6 (2.5)	5 (2.8)	1 (1.7)	0.618
Congenital anomalies	20 (8.5)	17 (9.7)	3 (5.0)	0.276
Presences of any chronic comorbidities	197 (83.5)	139 (79.0)	58 (96.7)	0.002
Presences of more than one comorbidities	105 (44.5)	79 (44.8)	26 (43.3)	0.741
Day of life at onset of VAP (day), median (IQR)	30.0 (19.0–48.5)	26.5 (17.0–44.5)	39.5 (26.5–55.5)	< 0.001
On antibiotic treatment at onset of VAP, n (%)	89 (37.7)	58 (32.9)	31 (51.7)	0.013
Use of TPN and/or intrafat, n (%)	174 (73.7)	134 (76.1)	40 (66.7)	0.175
Use of central venous catheter, n (%)	217 (91.9)	162 (92.0)	55 (91.7)	0.926
Clinical features, n (%)				
Fever	14 (5.9)	11 (6.3)	3 (5.0)	0.723
On HFOV/conventional ventilator	86 (36.4)/150 (63.6)	69 (39.2)/107 (60.8)	17 (28.3)/43 (71.7)	0.162
Septic shock	38 (16.1)	30 (17.0)	8 (13.3)	0.481
Metabolic acidosis	37 (15.7)	27 (15.3)	10 (16.7)	0.838
NTISS score at onset of VAP, median (IQR)	27.0 (25.0–29.0)	27.0 (25.0–29.0)	27.0 (24.0–29.0)	0.209
With concurrent bacteremia	44 (18.6)	35 (19.9)	9 (15.0)	0.689
Requirement of blood transfusion^a^	175 (74.2)	134 (76.1)	41 (68.3)	0.110
Requirement of high FiO_2_ (≥ 50%)^b^	96 (40.7)	75 (42.6)	21 (35.0)	0.167
Chest X-ray findings				0.597
New infiltrate	97 (41.1)	70 (41.7)	27 (38.9)	
Worsening infiltrate	126 (53.4)	95 (54.2)	31 (55.0)	
Persistent infiltrate	13 (5.5)	11 (4.2)	2 (6.0)	

*NSD* normal spontaneous delivery, *IQR* interquartile range, *HFOV* high-frequency oscillatory ventilator, *NTISS score* Neonatal Therapeutic Intervention Scoring System, *TPN* total parenteral nutrition

^a^Including leukocyte poor red blood cell and/or platelet transfusion

^b^To maintain SpO_2_ (pulse oximetry) > 94%

### Therapeutic responses and independent risk factors of treatment failure

Broad-spectrum antibiotics, namely, vancomycin or teicoplanin plus carbapenem or ceftazidime or cefotaxime were prescribed as empiric antibiotics for 80.1% of these VAP episodes, comparable in both monomicrobial and polymicrobial VAP episodes (Table 3). Although the percentage to receive inappropriate initial antibiotic therapy was relatively higher in the polymicrobial group than the monomicrobial VAP group (p = 0.054), neonates with polymicrobial lung infection were more susceptible to have modification of therapeutic antibiotics than the monomicrobial VAP group (63.3% vs. 42.6%, *P* < 0.001) after sputum culture and antimicrobial susceptibility testing results. The median (IQR) treatment duration of all VAP episodes was 9.8 (7.3–12.0) days. In this cohort, 23 (9.7%) patients died before discharge and the VAP attributable mortality rate of this cohort were 3.8% (9/236). The therapeutic responses were comparable between polymicrobial VAP episodes when compared with monomicrobial VAP episodes, both in the percentage of clinical resolution and microbiological resolution. A total of 36 VAP episodes were considered as treatment failure, including VAP attributable mortality (n = 9), death due to superinfection after VAP (n = 2), progression to bacteremia (n = 10), requirement of therapeutic antibiotics for more than two weeks (n = 17), and worsening of clinical symptoms after appropriate antibiotics for more than one week (n = 12).

**Table 3:** Therapeutic intervention and outcomes of all neonatal ventilator-associated pneumonia (VAP) in the CGMH, October 2017-March 2020

Characteristics	All VAP episodes (total n = 236)	Monomicrobial VAP episodes (total n = 176)	Polymicrobial VAP episodes (total n = 60)	P values
Therapeutic intervention, n (%)				
Initial empiric antibiotics				
Inappropriate initial antibiotics	74 (31.4)	49 (27.8)	25 (41.7)	0.054
Use of first line antibiotics	58 (24.6)	47 (26.7)	11 (18.3)	0.116
Use of broad-spectrum antibiotics	178 (75.4)	129 (73.3)	49 (81.7)	0.116
Modification of therapeutic antibiotics	120 (50.8)	82 (46.6)	38 (63.3)	< 0.001
Therapeutic antibiotics				
Use of first line antibiotics	72 (30.5)	52 (29.5)	20 (33.3)	0.627
Use of broad-spectrum antibiotics	164 (69.5)	124 (70.5)	40 (66.7)	0.627
Duration of antibiotic treatment (day), mean ± SD	9.6 ± 3.8	9.6 ± 3.8	9.8 ± 3.8	0.566
Therapeutic outcomes, n (%)				
Detailed clinical assessment, n (%)				0.503
Clinical resolution	91 (38.5)	66 (37.5)	25 (41.7)	
Delayed resolution	73 (30.9)	56 (31.8)	17 (28.3)	
Relapse or recurrent infection	20 (8.5)	15 (8.5)	5 (8.3)	
Superinfection	41 (17.4)	30 (17.0)	11 (18.3)	
Death	11 (4.7)	9 (5.1)	2 (3.3)	
Detailed microbial assessment, n (%)				0.462
Resolution	112 (47.5)	84 (47.7)	28 (46.7)	
Relapsed or recurrent infection	35 (14.8)	29 (16.5)	6 (10.0)	
Superinfection	72 (30.5)	49 (27.8)	23 (38.3)	
Clinical failure	17 (7.2)	14 (8.0)	3 (5.0)	
Overall clinical assessment, n (%)				0.414
Cure	200 (84.7)	147 (83.5)	53 (88.3)	
Treatment failure^a^	36 (15.3)	29 (16.5)	7 (11.7)	

*IQR* interquartile range

^a^Treatment failure was defined as neonates who required therapeutic antibiotics for more than two weeks, those progress to bacteremia, those with worsening clinical symptoms after appropriate antibiotics for one week, and neonates who died due to ventilator-associated pneumonia

We summarize the results of univariate and multivariate analyses of potential factors that were associated with treatment failure of VAP in this cohort (Table 4). Neither GA nor lower BBW were significantly associated with a higher risk of treatment failure. Treatment failure was not independently associated with any specific pathogens, MDR pathogens, polymicrobial VAP or inappropriate initial antibiotic treatment. After adjustment, independent risk factors for treatment failure in VAP were presences of concurrent bacteremia (OR 6.36; 95% CI 2.57–15.65, *P* < 0.001), septic shock (OR 3.14; 95% CI 1.22–8.17, *P* = 0.018), neonates on HFOV (OR 3.90; 95% CI 1.61–9.48, *P* = 0.003), and neonates with underlying neurological sequelae (OR 2.74; 95% CI 1.18–6.36, *P* = 0.019). The goodness-of-fit test of Hosmer and Lemeshow showed good agreement between observed and predicted values of the model (P = 0.65).

**Table 4:** Multivariate logistic regression analysis for independent risk factors of clinical treatment failure in neonatal ventilator-associated pneumonia

Variables	Univariate analysis		Multivariate analysis	
	OR (95% CI)	P values	Adjusted OR (95% CI)	P values
Gestational age				
< 26 weeks	1.24 (0.37–4.17)	0.725		
26–28 weeks	1.35 (0.42–4.36)	0.617		
29–33 weeks	1 (reference)			
≥ 34 weeks	1.93 (0.42–8.84)	0.395		
Septic shock	4.67 (2.11–10.33)	< 0.001	3.14 (1.22–8.13)	0.018
On HFOV vs. conventional ventilator	4.45 (2.09–9.47)	< 0.001	3.90 (1.61–9.48)	0.003
Inappropriate initial antibiotics	0.74 (0.31–1.80)	0.513		
Polymicrobial VAP	0.67 (0.28–1.62)	0.373		
MDR pathogens associated VAP	1.05 (0.51–2.16)	0.896		
Presences of neurological sequelae	2.53 (1.22–5.23)	0.012	2.74 (1.18–6.36)	0.019
Bronchopulmonary dysplasia	0.75 (0.35–1.57)	0.440		
Severity of illness at onset of VAP				
Every 3 increase in NTISS scores	3.01 (1.44–6.30)	0.003	2.16 (0.93–5.00)	0.074
Concurrent sepsis	5.73 (2.64–12.38)	< 0.001	6.34 (2.57–15.65)	< 0.001
Thrombocytopenia	3.00 (1.24–7.27)	0.015	1.50 (0.52–4.34)	0.452

*HFOV* high frequency oscillatory ventilator, *OR* odds ratio, *95% CI* 95% confidence interval, *MDR* multidrug resistant, *NTISS* Neonatal Therapeutic Intervention Scoring System

## Discussion

The strict diagnostic criteria of CDC neonatal VAP were applied in this study to investigate the clinical and epidemiological features of polymicrobial VAP in the NICU. We found that 25.4% of neonatal VAP episodes were polymicrobial VAP episodes, which were more likely to occur in neonates with long-term intubation and underlying chronic comorbidities, especially BPD. Most of the clinical characteristics were not statistically different between polymicrobial versus monomicrobial VAP episodes. However, MDR pathogens (particularly *Pseudomonas aeruginosa* or MRSA) were more likely to be involved in polymicrobial VAP episodes than in monomicrobial VAP episodes, which has never been identified in previous studies [2, 4, 27, 28]. We found that the severity of VAP and underlying chronic comorbidities were independently associated with treatment outcomes.

We found the percentage of MDR pathogens in our series were significantly higher than previous studies [1, 7, 27, 28], especially in polymicrobial VAP cases and neonates with multiple chronic comorbidities. In our cohort, a lot of the *E coli* and *K. pneumonia* strains from endotracheal aspirates were MDR pathogens, but most *Pseudomonas aeruginosa* strains were surprisingly susceptible to gentamicin and/or third-generation cephalosporin. This is in contrast to our previous studies of MDR gram-negative bacteremia in the NICU, but is consistent with previous studies of adult VAP [13, 29, 30]. These antibiotic-resistant pathogens were selected by previous empiric and therapeutic antibiotics, because vancomycin or teicoplanin plus gentamicin or third-generation cephalosporin is frequently prescribed in our NICU for neonatal late-onset sepsis or catheter-related bloodstream infections [31, 32]. Our previous studies have documented that previous antibiotic exposure, especially broad-spectrum antibiotics, is significantly associated with emergence of MDR pathogens [23, 29]. Therefore, MDR gram-negative bacilli and MRSA are more likely to be the long-term colonized pathogen in the endotracheal tube after antibiotic selection and account for the majority of neonatal VAP [33]. Because emergence of MDR pathogens has been the major issue in the ICU, we suggest that NICU surveillance and more epidemiological data may be required for better development of optimized therapeutic strategies, which can be guided by this information and the patient’s risk factors for MDR pathogens [32, 34, 35].

Both Infectious Diseases Society of America and American Academy of Pediatrics suggest that broad-spectrum antibiotics can be considered only in high risk patients with severe sepsis or septic shock, or in those with a high risk of clinical deterioration or multi-organ failure [36, 37]. In our cohort, initial broad-spectrum antibiotics were prescribed in 75.4% episodes of these VAP episodes because these patients were considered to have high risks of infection caused by MDR pathogens, including long-term hospitalization, previous antibiotic exposure, presence of CVC in situ, and multiple comorbidities. In addition, clinicians cannot take the risk of clinical deterioration or progression to severe sepsis or septic shock since 86 (36.4%) of the VAP episodes occurred in neonates on HFOV treatment and approximately three-fourth of these VAP episodes had a high NTISS score > 25, indicating the high severity of illness. Although we found that inappropriate initial antibiotic therapy did not significantly contribute to worse outcomes, it was only 31.4% of these cases and we cannot underestimate the importance of initial appropriate treatment. Additionally, the influences of MDR pathogens and polymicrobial VAP episodes may also be masked by the high percentage of empirical broad-spectrum antibiotic therapy. Overuse of broad-spectrum antibiotics has now become as an important issue and contributed to emergence of MDR pathogens, which require more prescription of broad-spectrum antibiotics and create a vicious circle [32, 38, 39].

No data are available regarding the clinical characteristics and therapeutic outcomes of neonates with polymicrobial VAP in the literature. Polymicrobial VAP is supposed to cause therapeutic challenge and increased use of antibiotics in the NICU because all isolated microorganisms have to be covered. In this series, only more modification of therapeutic antibiotics was documented. In adult studies of nosocomial VAP, the mortality rate between monomicrobial and polymicrobial VAP episodes were comparable [13, 40]. We found neonatal VAP rarely progressed to systemic bacteremia or have rapid deterioration, even in cases of inappropriate initial antibiotic therapy. This accounts for the lower attributable mortality rate of neonatal VAP when compared with neonatal bacteremia or adult VAP [29, 40]. Therefore, it is worth reconsidering the necessity of using broad-spectrum antibiotics to treat neonates with VAP that without concurrent bacteremia.

The reported incidence rate of neonatal VAP is 2.7–10.9 cases/1000 mechanical ventilation days in developed countries and 32–37.2 cases/1000 ventilator days in developing countries [1–3, 7]. The incidence rate of VAP in this cohort was underestimate because we did not count the repeated episodes or recurrent episodes of VAP. In this study, nonquantitative cultures of the endotracheal aspirate were used for diagnoses of neonatal VAP, which is commonly used in clinical practice in the NICU because quantitative culture of the BAL fluid is often unavailable in extremely preterm or low birth weight neonates [41]. The incidence rate of VAP in our cohort was comparable with previous studies and most of them applied similar diagnostic criteria for neonatal VAP [1, 3, 7]. Therefore, it is clinically applicable to consider all potential microorganisms regardless of the number of CFU per milliliter as the causative pathogens of VAP [1, 9].

There are some limitations in this study. Although we applied the updated and strict diagnostic criteria and prospectively followed these cases, this was an observational, non-interventional study and we cannot conclude whether therapeutic policies will affect the outcomes or not. Therefore, a future randomized controlled trial is required to reach clear conclusion regarding this issue. A high proportion of VAP cases were treated with broad-spectrum antibiotics in our tertiary level NICUS, which may be due to higher illness severity and more chronic comorbidities in our cohort. Therefore, we cannot find significant impacts of MDR pathogens or polymicrobial VAP on the outcomes. This is a single center study from tertiary level medical center and these results are less applicable to nonteaching hospitals. In addition, the sample size of this study is only moderate and we did not enroll the repeated or recurrent episodes of VAP for analysis. However, the prospective study design, strict and uniform criteria of neonatal VAP, close observation and complete follow-up of all cases without any missing data are the major strengths of this study.

## Conclusion

In conclusion, polymicrobial VAP is not uncommon in the NICU, and commonly occurred in extremely preterm neonates with prolonged ventilation and underlying bronchopulmonary dysplasia. Although the therapeutic responses and outcomes were not significantly worse when compared with monomicrobial VAP, polymicrobial VAP episodes were more often associated with MDR pathogens and requirement of antibiotic modification. Overuse of broad-spectrum antibiotics has emerged as an important issue in the NICU, so it is urgently needed to consider antibiotic stewardship programs for neonatal VAP. Although neonatal VAP is not associated with a high attributable mortality rate, significant morbidity is noted in neonates with VAP, especially those with concurrent bacteremia, higher severity of illness and underlying neurological sequelae.

## Supplementary Information

**Additional file 1: Table S1.** Diagnostic criteria for neonatal ventilator-associated pneumonia in this study, based on the CDC criteria [1].

## Data Availability

The datasets used/or analyzed during the current study available from the corresponding author on reasonable request.

## Abbreviations

NBBAL: Nonbronchoscopic bronchoalveolar lavage
CDC: Centers for Disease Control and Prevention
CI: Confidence interval
CoNS: Coagulase negative Staphylococcus
CVC: Central venous catheter
CGMH: Chang Gung Memorial Hospital
GNB: Gram-negative bacteremia
HAIs: Healthcare associated infections
LOD: Late-onset disease
MDR: Multidrug-resistant
MDR VAP: MDR associated VAP
NTISS: Neonatal Therapeutic Intervention Scoring System
OR: Odds ratio
TPN: Total parenteral nutrition
VAP: Ventilator-associated pneumonia
