# Supplementary material for: Clinical characteristics and outcomes of neonates with polymicrobial ventilator-associated pneumonia in the intensive care unit
Source: BMC Infect Dis. 2021 Sep 17;21:965. doi: 10.1186/s12879-021-06673-9 (PMC8446475; doi:10.1186/s12879-021-06673-9)
Supplement: Supplementary file 1 — Additional file 1: Table S1. Diagnostic criteria for neonatal ventilator-associated pneumonia in this study, based on the CDC criteria [1]. [file 12879_2021_6673_MOESM1_ESM.docx]

Table S1. Diagnostic criteria for neonatal ventilator-associated pneumonia in this study, based on the CDC criteria [1]

| Radiological signs |  | Patients with one or more (in patients with underlying diseases two or more) chest X-rays with one of the following findings: |
| --- | --- | --- |
|  |  | -new or progressive and persistent infiltrate |
|  |  | -consolidation |
|  |  | -cavitation |
|  |  | -pneumatoceles |
| Clinical signs and symptoms |  | Worsening of gas exchange [e.g. oxygen desaturations (e.g. pulse oximetry < 94%), increased oxygen requirements, or increased ventilation demand] and three of the following: |
|  |  | -temperature instability with no other recognized cause |
|  |  | -leukopenia ( < 4,000 WBC/mm^3^) or leukocytosis ( > 15,000 WBC/mm^3^) and left shift ( > 10% band forms) |
|  |  | -new onset of purulent sputum, or change in the character of sputum, or increase in respiratory secretions, or increased suctioning requirements. |
|  |  | -apnea, tachypnea, nasal flaring with retraction of chest wall or grunting |
|  |  | -wheezing, rales, or rhonchi |
|  |  | -cough |
|  |  | -bradycardia ( < 100 beats/min) or tachycardia ( > 170 beats/min) |
| Microbiological findings |  | At least one of the followings: |
|  |  | -positive growth in blood culture not related to another source of infection |
|  |  | -positive growth pleural fluid culture |
|  |  | -positive quantitative culture from a minimal contaminated low respiratory tract specimen [e.g. BAL (≥10^4^ CFU/ml) or protected specimen brushing (≥10^3^ CFU/ml)] |
|  |  | -≥5% BAL-obtained cells contain intracellular bacteria on direct microscopic examination (e.g. Gram stain) |
|  |  | -histopathological exam shows at least one of the following criteria for pneumonia  abscess formation or foci of consolidation with intense PMN accumulation in bronchioles and alveoli;  positive quantitative culture of lung parenchyma (≥10^4^ CFU/g tissue), or evidence of lung parenchyma invasion by fungal hyphae or pseudohyphae |

WBC: white blood cells; CFU: colony-forming units.
